# *RASSF2*, a potential tumour suppressor, is silenced by CpG island hypermethylation in gastric cancer

**DOI:** 10.1038/sj.bjc.6602854

**Published:** 2005-11-01

**Authors:** M Endoh, G Tamura, T Honda, N Homma, M Terashima, S Nishizuka, T Motoyama

**Affiliations:** 1 Department of Pathology, Yamagata University School of Medicine, 2-2-2 Iida-nishi, Yamagata 990-9585, Japan; 2 Department of Surgery, Fukushima Medical University, 1 Hikarigaoka, Fukushima 960-1295, Japan; 3 Molecular Therapeutics Program, Center for Cancer Research, National Cancer Institute, National Institutes of Health, Bethesda, MD 20892, USA

**Keywords:** *RASSF2*, hypermethylation, gastric cancer

## Abstract

*RASSF2*, a member of the *RASSF1* family, has recently been identified as a potential tumour suppressor. We examined methylation status in multiple regions which included the CpG island and spanned the transcription start site of *RASSF2* in 10 gastric cancer cell lines, as well as 78 primary gastric cancers and corresponding non-neoplastic gastric epithelia. Hypermethylation of *RASSF2* in at least one of the regions examined was detected in seven (70%) of the 10 cell lines; two (20%) exhibited hypermethylation in all the regions examined including the transcription start site and lost expression of *RASSF2* mRNA, which could, however, be restored by 5-aza-2′ deoxycytidine treatment, while the other five (50%) cell lines exhibited hypermethylation at the 5′- and/or 3′- edge, with four of them expressing *RASSF2* mRNA. In primary gastric cancers and corresponding non-neoplastic gastric epithelia, frequencies of *RASSF2* methylation ranged from 29% (23 out of 78) to 79% (62 out of 78) and 3% (two out of 78) to 60% (47 out of 78), respectively, at different CpG sites examined. Methylation was frequently observed at the 5′- and 3′- edges, and became less frequent near the transcription start site in both the primary gastric cancers and corresponding non-neoplastic gastric epithelia. Hypermethylation near the transcription start site was mostly cancer-specific. We thus showed that *RASSF2* is silenced by hypermethylation near the transcription start site in gastric cancer. Hypermethylation was found initially to occur at the 5′- and 3′- furthest regions of the CpG island in non-neoplastic gastric epithelia, to gradually spreads near the transcription start site to shut down *RASSF2* expression, and ultimately to constitute a field-defect placing tissue increased risk for development of gastric cancer.

Human cancers develop and progress by accumulation of both genetic and epigenetic alterations. Mutations of *p53* are frequent genetic (structural) alterations in gastric cancer (Tamura, 2002). *E-cadherin* is inactivated through any combinations of gene mutation, loss of heterozygosity (LOH), and promoter hypermethylation in gastric cancer, especially that of undifferentiated histological type (Tamura *et al*, 2001). Recent evidences of frequent silencing of *hMLH1*, *p16*, *RUNX3*, and other tumour suppressor and tumour-related genes by promoter hypermethylation suggests that epigenetic alterations play the most important role in gastric carcinogenesis (Tamura, 2004). Furthermore, promoter hypermethylation initially occurs in non-neoplastic gastric epithelia, increases with age, and ultimately silences gene function to constitute a field-defect that may predispose tissues to development of gastric cancer (Waki *et al*, 2002, 2003b). Many genes become methylated in gastric epithelia during aging (Waki *et al*, 2003a), although frequencies of methylation depends on the sites of CpGs examined within a gene promoter (Satoh *et al*, 2002).

Ras family genes are associated with the signal transduction from G-protein-coupled receptors and activation of the ras-signal transduction pathway frequently observed in human tumours (Shih *et al*, 1982; Malumbres and Pellicer, 1998; Ayllon and Rebollo, 2000). Ras proteins interact with a wide spectrum of regulators and downstream effectors to produce various cellular responses, including cell proliferation, differentiation, and apoptosis (Malumbres and Pellicer, 1998). Recently, the ras effectors/tumour suppressors *RASSF1* and *NORE1* were found to be inactivated by promoter hypermethylation in a variety of human tumours (Vavvas *et al*, 1998; Dammann *et al*, 2000; Pfeifer *et al*, 2002; Hesson *et al*, 2003; Vos *et al*, 2003b). *RASSF2* was identified as a third member of the *RASSF1* family and a ras effector/tumour suppressor (Vos *et al*, 2003a). RASSF2 binds to K-ras in a GPT-dependent manner via the Ras effector domain, but interacts with H-ras weakly (Vos *et al*, 2003a). *RASSF2* inhibits the growth of tumour cells, promotes both cell cycle arrest and apoptosis, and is frequently downregulated in lung tumour cell lines (Vos *et al*, 2003a). The *RASSF2*-methylated RKO, colorectal cell line, without *RASSF2* RNA expression showed increase of apoptosis and cell growth inhibition (Akino *et al*, 2005). These observations indicate that *RASSF2* shows tumour suppressor activity in various human cancers.

In the present study, we examined the methylation status of *RASSF2* at multiple regions which included the CpG island and spanned the transcription start site in gastric cancer cell lines, as well as primary gastric cancers and corresponding non-neoplastic gastric epithelia. The regions critical for methylation silencing of *RASSF2* mRNA expression were examined by analyses of cell lines, while methylation spreading near the *RASSF2* transcription start site was examined by analyses of primary gastric cancers and corresponding non-neoplastic gastric epithelia.

## MATERIALS AND METHODS

### Samples

A total of 10 gastric cancer cell lines with various histologies were cultured under appropriate conditions in our laboratory: MKN1, an adenosquamous cell carcinoma; MKN7, a well-differentiated adenocarcinoma; MKN28 and MKN74, moderately differentiated adenocarcinomas; MKN45 and KWS-I, poorly differentiated adenocarcinomas; KATO-III, a signet ring cell carcinoma; ECC10 and ECC12, endocrine cell carcinomas; and TSG 11, a hepatoid carcinoma. In total, 78 gastric cancer samples and matching non-neoplastic gastric tissues were obtained at surgery from 78 patients. Clinicopathological data were available for 71 of the 78 patients (Table 1). The patients ranged in age from 43 to 89 years (mean, 67.6 years). All the patients received follow-up care, for a median of 36.1 months (range, 1–77 months.). All samples were stored at −80°C until processed. DNA was extracted from the 10 gastric cancer cell lines, 78 primary gastric cancers and corresponding non-neoplastic gastric epithelia using SepaGene (Sankyo-Junyaku, Tokyo, Japan). Total RNA was isolated from the 10 gastric caner cell lines with the TRIZOL reagent (Gibco BRL, Life Technologies, Gaithersburg, MD, USA).

### Methylation-specific PCR (MSP)

**Figure 1:**
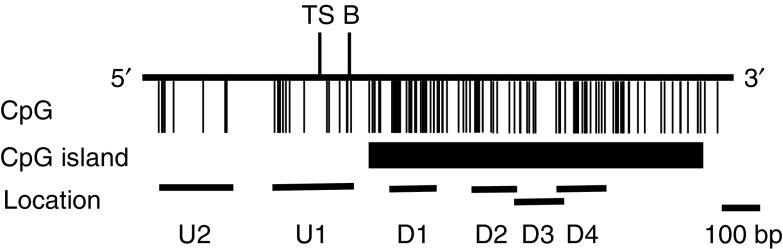
Location of methylation-specific PCR (MSP) near the transcription start site including (**A**) CpG island of *RASSF2*. Vertical lines indicate individual CpG sites. TS, transcription start site; (**B**) exon 1 and intron 1 boundary.

Treatment of DNA samples with sodium bisulphite converts all unmethylated cytosines to uracils but does not affect methylated cytosines. Briefly, 2 *μ*g of genomic DNA was denatured by treatment with NaOH and modified by sodium bisulphite. The samples were then purified using Wizard DNA purification resin (Promega, Madison, WI, USA), treated with NaOH, recovered in ethanol and resuspended in 30 *μ*l of distilled water. Amplification was performed in a 20 *μ*l reaction volume containing 2 *μ*l of GeneAmp PCR Gold Buffer (PE Applied Biosystems, Foster City, CA, USA), 1.0 mM MgCl_2_, 1 *μ*l of each primer, 0.2 *μ*M dNTPs, and 1 U of *Taq* polymerase (Ampli*Taq* Gold DNA Polymerase, PE Applied Biosystems). Hot start PCR was performed in a thermal cycler (GeneAmp 2400, PE Applied Biosystems) for 35 cycles, each of which consisted of denaturation at 95°C for 15 s, annealing at 55°C for 15 s, and extension at 72°C for 30 s, followed by a final 7-min extension at 72°C. A positive control and a negative control (distilled water without DNA) were included for each amplification. The PCR products were separated on a 6% nondenaturing polyacrylamide gel. To detect regions playing critical roles in regulating the expression of *RASSF2A* mRNA, we designed six primer sets spanning the *RASSF2* transcription start site and including the CpG island (GenBank accession number AL 133354) (Figure 1). The following primer sets were used: 5′-GGT TTA AGT TTT TCG GTT TAT TC-3′ and 5′-CAC GTC TAA CCG ACC CGC CAA ATC G-3′ for the methylated *RASSF2*-U2 sequence (upstream 2; the upstream sequence furthest from the transcription start site) (212 bp); 5′-GGT TTA AGT TTT TTG GTT TAT TTG GA-3′ and 5′-TCA CAT CTA ACC AAC CCA CCA AAT CA-3′ for the unmethylated *RASSF2*-U2 sequence (213 bp); 5′-GTT TTT ATC GGA TTT GTT CGT TC-3′ and 5′-CCA ACC CGA AAA AAT CGC TAA CGA CG-3′ for the methylated *RASSF2*-U1 sequence (upstream 1; the upstream sequence nearest the transcription start site) (237 bp); 5′-GTT TTT ATT GGA TTT GTT TGT TTG-3′ and 5′-CCA ACC CAA AAA AAT CAC TAA CAA CA-3′ for the unmethylated *RASSF2*-U1 sequence (237 bp); 5′-GTA TTT CGC GTT AGT GTT TC-3′ and 5′-TTA AAC CCG ACC CGC CGA TCG-3′ for the methylated *RASSF2*-D1 sequence (downstream 1; the downstream sequence nearest the transcription start site in CpG island) (183 bp); 5′-TTG GGT ATT TTG TGT TAG TGT TTT GTT-3′ and 5′-ATT TAA ACC CAA CCC ACC AAT CAA T-3′ for the unmethylated *RASSF2*-D1 sequence (189 bp); 5′-CGG GTT TAA AAA GAA GGA AGG AC-3′ and 5′-GCG CGA ACC CCC GCC AAA AAC CG-3′ for the methylated *RASSF2*-D2 sequence (135 bp); 5′-GTG GGT TTA AAA AGA AGG AAG GAT-3′ and 5′-CAC ACA AAC CCC CAC CAA AAA CCA T-3′ for the unmethylated *RASSF2*-D2 sequence (137 bp); 5′-TTC GTT TAG AAG ACG GCG GC-3′ and 5′-CCT TCC TTC TTT TTA AAC CCG-3′ for the methylated *RASSF2*-D3 sequence (166 bp); 5′-AAT TTG TTT AGA AGA TGG TGG T-3′ and 5′-ATC CTT CCT TCT TTT TAA ACC CA-3′ for the unmethylated *RASSF2*-D3 sequence (170 bp); 5′-CGA AGG AGG GCG GGG AGA TC-3′ and 5′-TCC GCC GCC GTC TTC TAA ACG-3′ for the methylated *RASSF2*-D4 sequence (downstream 4; the downstream sequence of furthest from the transcription start site) (148 bp); and 5′-GTT TTG AAG GAG GGT GGG GAG ATT-3′ and 5′-AAT CCA CCA CCA TCT TCT AAA CA-3′ for the unmethylated *RASSF2*-D4 sequence (154 bp). The positions of amplified regions are shown in Figure 1. As a positive control, Sss I methylase (New England BioLabs, Inc., Beverly, MA, USA) was used to methylate 100 mg of liver tissue-derived DNA obtained from an autopsy sample, and was modified by sodium bisulphite as described above.

### Reverse transcription–PCR (RT–PCR)

Isolated RNA was reverse-transcribed and amplified using a one-step RT–PCR System (Gibco BRL). Primer sequences used were 5′-AAG ACA TCC GTG TTC ACA CC-3′ and 5′-TCG TTC TCA TGG CTC AGA TT-3′ for *RASSF2A* mRNA (462 bp); and sense, 5′-AAA TCT GGC ACC ACA CCT T-3′ and antisense, 5′-AGC ACT GTG TTG GCG TAC AG-3′ for *β*-actin (646 bp). Primers for *RASSF2A* mRNA can also amplify *RASSF2B* mRNA product, but product size of *RASSF2B* mRNA (409 bp) is smaller than that of *RASSF2A* mRNA. Thus, they could be differentiable.

### 5-aza-2′-deoxycytidine (5-aza-dC) treatment

To examine restoration of *RASSF2* mRNA expression, two cell lines, KATO-III and KWS-I, were incubated for 96 h with 5 *μ*M 5-aza-dC (Sigma, St Louis, MO, USA), and then harvested for RNA extraction and RT–PCR. MKN74, which expresses *RASSF2* mRNA with an unmethylated CpG island, was used as a control.

### Statistical analysis

Statistical comparisons were performed using Fisher's exact probability test and Mann–Whitney's *U*-test. Values of *P*<0.05 were considered significant.

## RESULTS

### Methylation status and mRNA expression of RASSF2 in gastric cancer cell lines

**Figure 2:**
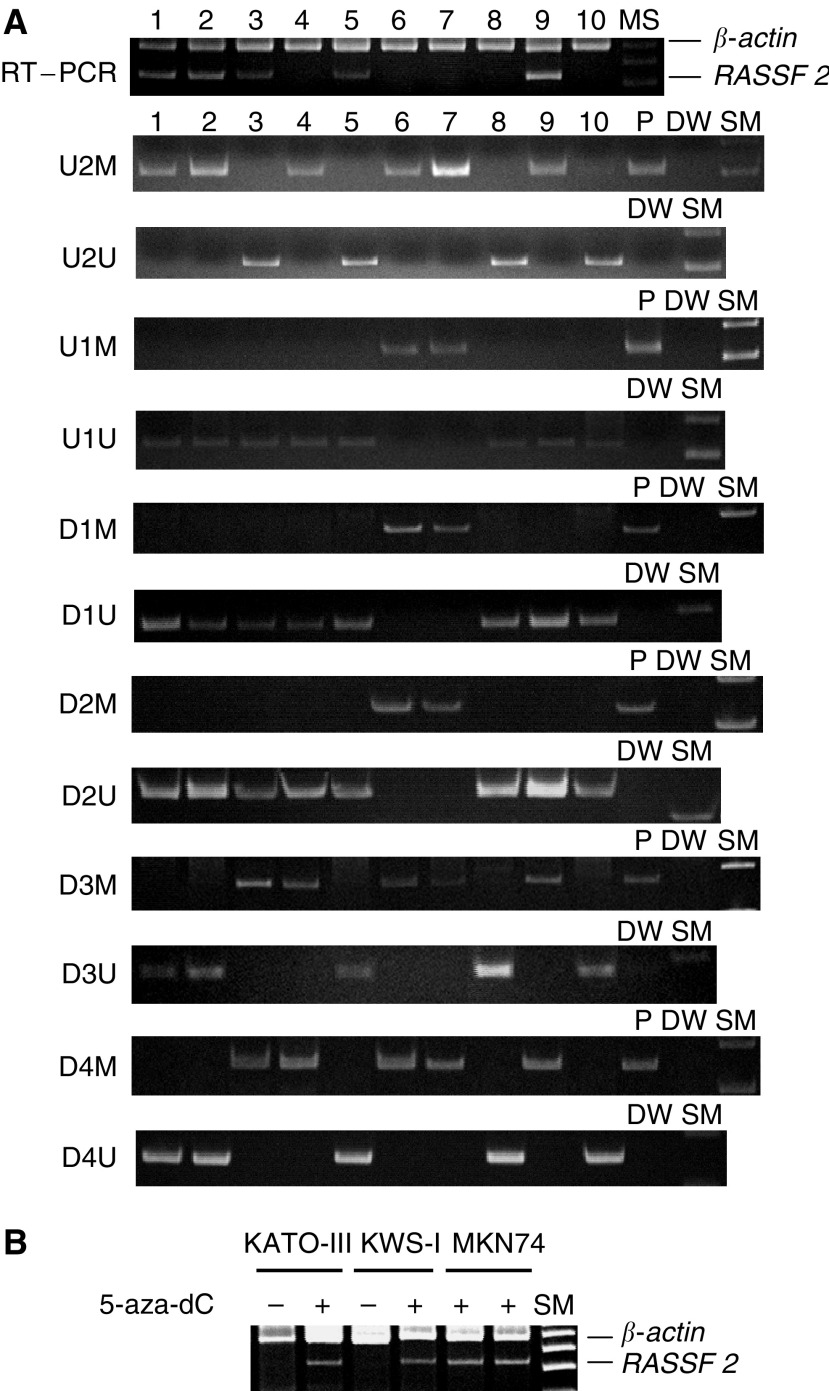
Reverse transcription–PCR and methylation-specific PCR (MSP) (**A**), and comparison of *RASSF2* mRNA expression before (−) and after (+) 5-aza-dC treatment (**B**) in gastric cancer cell lines. (**A**) *RASSF2* mRNA is present in lanes 1–3, 5, and 9. Methylated-PCR products from U2M to D4M are present at all the regions in lanes 6 and 7, at only the 5′- and/or 3′- edge in lanes 1–4, and 9, and not present in lanes 5, 8, and 10. Unmethylated-PCR products from U2U to D4U are alternatively present in lanes 1–5, and 8–10, and not present in lanes 6 and 7. Lanes 1, MKN1; 2, MKN7; 3, MKKN28; 4, MKN45; 5, MKN74; 6, KATO-III; 7, KWS-I; 8, TSG11; 9, ECC10; 10, ECC12; P, positive control; DW, distilled water; and SM, size marker. (**B**) Treatment with 5-aza-dC restored *RASSF2* mRNA expression in KATO-III and KWS-I, but did not affect levels of *RASSF2* expression in MKN74.

Hypermethylation of *RASSF2* in at least one of the regions examined was detected in seven of the 10 cell lines, MKN1, MKN7, MKN28, MKN45, KATO-III, KWS-I, and ECC10 (Figure 2A). Among these, KATO-III and KWS-I exhibited hypermethylation in all the regions examined, and lost *RASSF2* mRNA expression (Figure 2A), which, however, was restored by 5-aza-dC treatment (Figure 2B). The other five of these seven cell lines, MKN1, MKN7, MKN28, MKN45, and ECC10 exhibited methylation only in outskirt regions, and four of them expressed *RASSF2* mRNA (Figure 2A). Three cell lines, MKN74, TSG11, and ECC12, did not exhibit hypermethylation in any region. TSG11 and ECC12 lost *RASSF2* mRNA expression despite being unmethylated.

### Hypermethylation RASSF2 CpG island in primary gastric cancer and corresponding non-neoplastic gastric epithelia

**Figure 3:**
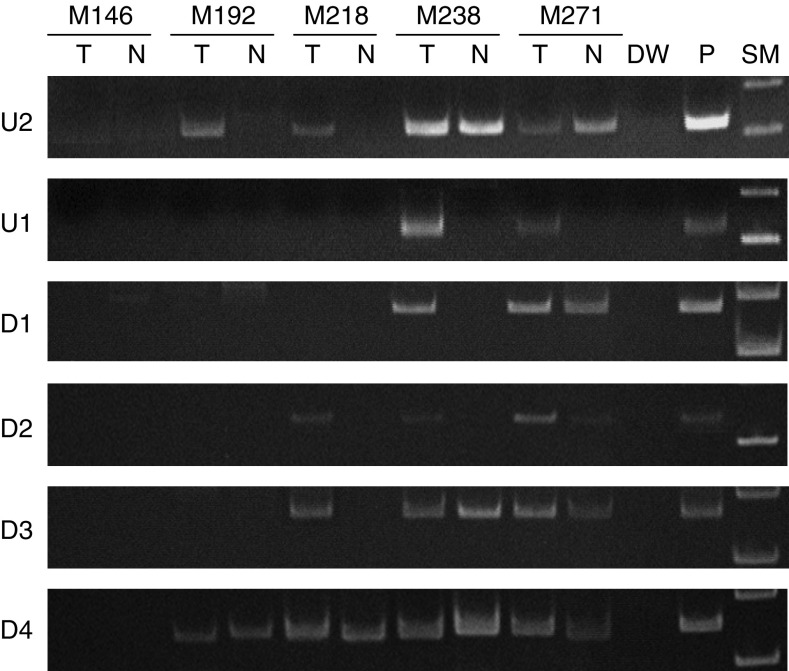
Examples of methylation-specific PCR (MSP) in primary gastric cancers (T) and corresponding non-neoplastic gastric epithelia (N). Methylation is frequent in outskirt regions (U2 and D4), but becomes less frequent near the transcription start site (U1 and D1) in both gastric cancers and non-neoplastic gastric epithelia. DW, distilled water; P, positive control; and SM, size marker.

**Figure 4:**
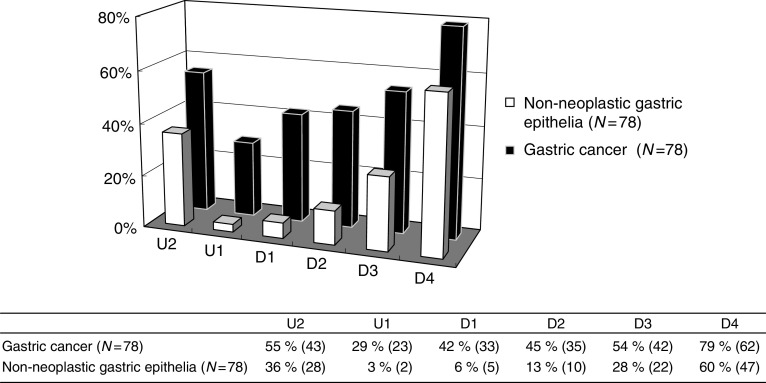
Percentages of hypermethylation in various regions (U2–D4) of *RASSF2*.

Methylation frequencies of *RASSF2* varied in the regions upstream and downstream of the transcription start site. Of primary gastric cancers and corresponding non-neoplastic epithelia, hypermethylation was observed in 55% (43 out of 78) and 36% (28 out of 78) at U2, 29% (23 out of 78) and 3% (two out of 78) at U1, 42% (33 out of 78) and 6% (five out of 78) at D1, 45% (35 out of 78) and 13% (10 out of 78) at D2, 54% (42 out of 78) and 28% (22 out of 78) at D3, 79% (62 out of 78) and 60% (47 out of 78) at D4, respectively (Figures 3 and 4). Unmethylated RASSF2 sequence was present in all the samples (data not shown). Gastric cancers with methylation at U1 and D1 exhibited significantly less frequent lymphatic permeation than unmethylated gastric cancers (Table 1) No significant correlation was observed between methylation status of *RASSF2* and other clinicopathological factors. Methylation status of *RASSF2* did not significantly influence event-free survival rate, as determined by Kaplan–Meier curve analysis with the log-rank test and Breslow–Gehan–Wilcoxon test (data not shown).

## DISCUSSION

Results in gastric cancer cell lines suggest that hypermethylation near the transcription start site was associated with loss of *RASSF2* mRNA expression, although other mechanisms might also contribute to *RASSF2* silencing. While by analyses of samples from primary gastric cancer patients, frequencies of methylation at each region investigated were higher in primary gastric cancers than in corresponding non-neoplastic gastric epithelia. Very recently, Akino *et al* reported the significant association between methylation and expression status of *RASSF2* in colon cancer cell lines and primary colorectal cancers. They identified the *RASSF2* promoter region and showed that absence of *RASSF2* transcription was caused by DNA methylation, not by alteration of transcription factors, and also showed histone acetylation at the 5′ region of *RASSF2* in colorectal cancer cell lines with DNA methylation (Akino *et al*, 2005). It has also been reported that inactivation of *RASSF2* with siRNA increased phospholilation of MAP kinase, and that *RASSF2* suppressed downstream signalling in the RAS pathway (Akino *et al*, 2005). Colorectal tumours with *RASSF2* methyaltion showed *K-ras*/*BRAF* mutations significantly more frequently than those without *RASSF2* methyaltion (Akino *et al*, 2005), although earlier studies have showed that *K-ras* mutation and *RASSF2* methylation are mutually exclusive (Hesson *et al*, 2005). In the present study, we have showed that *RASSF2* methylation is frequent in gastric cancer, despite the rarity of *K-ras*/*BRAF* mutations in this tumour (Kim IJ *et al*, 2003).

Methylation in the 5′- and 3′- furthest regions within the CpG island was very frequent in both primary gastric cancers and corresponding non-neoplastic gastric epithelia, but became less frequent near the transcription start site. Methylation near the transcription start sites at U1 and D1 appeared to be mostly cancer-specific. These findings are essentially the same as those for *DAP-kinase* hypermethylation (Satoh *et al*, 2002). Therefore, the pattern of spread of methylation, initially at the outskirts of CpG islands and then progressing to regions critical for gene silencing, might be common to various types of methylation-related gene silencing. Since frequencies of methylation are influenced by the location of MSP, as noted above, the significance of detection of DNA methylation in a giver tumour suppressor or tumour-related gene varied significantly. For example, hypermethylation near the transcription start site, which is cancer-specific and results in gene silencing, can be used as a diagnostic marker of malignancy in tissues or other samples, such as serum or ascites, and hypermethylation at a region next to such a critical region might be an early signal of carcinogenesis.

Gastric cancers with methylation at U1 and D1, a change critical for *RASSF2* silencing, exhibited significantly less frequent lymphatic permeation than unmethylated gastric cancers. Gastric cancers with a high frequency of microsatellite instability (MSI-H), which is the result of *hMLH1* silencing by hypermethylation, exhibited less frequent lymph node metastasis (dos Santos *et al*, 1996; Wu *et al*, 2000), and several genes are simultaneously methylated together with *hMLH1* (Kim H *et al*, 2003; Homma *et al*, 2005).

In conclusion, hypermethylation of *RASSF2* initially occurs in the 5′- and 3′- outskirt regions of the CpG island in non-neoplastic gastric epithelia, spreads near the transcription start site to shut down *RASSF2* expression, and ultimately constitutes a field-defect which places tissue at increased risk for the development of gastric cancer.

## Figures and Tables

**Table 1:** Correlation between RASSF2 CpG island hypermethylation and clinicopathological characteristics in gastric cancer

	***RASSF2* methylation status**		
**Characteristics**	**Methylated at U1and D1**	**Others**	***P*-value**
Number of patients	21	50	
Age (mean)	69.4	66.9	NS
*Gender*			NS
M	15	36	
F	6	14	
			
*Stage*			NS
Early	7	8	
Advanced	14	42	
			
*Histological differentiation*			NS
Differentiated	12	27	
Undifferentiated	9	23	
			
*Location*			NS
Upper	4	9	
Middle	6	18	
Lower	10	19	
Unknown	1	4	
			
*Lymph node metastasis*			NS
Present	11	31	
Absent	10	19	
			
*Lymphatic permeation*			0.033
ly (−)	7	6	
ly (+)	14	44	
			
*Venous permeation*			NS
v (−)	8	10	
v (+)	13	40	

NS=not significant by Fisher's exact probability test and Mann–Whitney's *U*-test.
